# Investigating relationship between religious commitment and moral sensitivity in nurses working in ICU

**DOI:** 10.1186/s13104-020-4912-x

**Published:** 2020-01-28

**Authors:** Ebrahim Khalighi, Laleh Solaimanizadeh, Milad Borji, Asma Tarjoman, Behrouz Soltany, Farzad Zareie

**Affiliations:** 1 Department of Anesthesiology, Medicine Faculty, Ilam University of Medical Science, Ilam, Iran; 2 Department of Nursing, Faculty of Nursing and Midwifery, Bam University of Medical Science, Bam, Iran; 3 Department of Nursing, Faculty of Nursing and Midwifery, Kermanshah University of Medical Science, Kermanshah, Iran; 4 Student Research Committee, Kermanshah University of Medical Sciences, Kermanshah, Iran

**Keywords:** Religious commitment, Moral sensitivity, Nurse

## Abstract

**Objectives:**

This study aimed at determining the relationship between religious commitment and moral sensitivity among nurses working in the ICU sections in the west of Iran. The present study was a cross-sectional descriptive-analytic study carried out on nurses working in ICU wards of two western cities in Iran. The instrument used includes a demographic questionnaire, religious commitment questionnaire and moral sensitivity questionnaire in nurses. First, the researchers referred to the ICU wards of the hospitals in the cities after receiving permission from the relevant authorities by referring to three shifts in the morning, evening and night shifts and holidays. The researchers, while explaining the research goals for the nurses participating in the study, obtained their informed consent to participate in this study.

**Results:**

According to the findings, mean (SD) of the overall score of religious commitment was equal to 36.38 (4.58) and mean (SD) of MS score of nurses was 59.21 (12.65). Also, 91 nurses (82.7%) had average MS, 7 (6.4%) had low MS and 12 (10.9%) had high MS.

## Introduction

Moral sensitivity causes the nurse to better manage and treat patients in relation to the therapeutic and non-therapeutic measures [1]. Moral sensitivity makes the nurse susceptible to ethical issues in the professional environment and, by increasing the amount of power of diagnosing ethics issues, obtains the skill required to acquire problem-solving skills in ethical dilemmas [2, 3].

Religious commitment means thinking, feeling and behaving in accordance with the beliefs and teachings of a religion [4, 5]. In fact, it can be said that religious commitment shows how religion is in the personal and social life of man. Religious commitment also reflects respect for values and adherence to beliefs in everyday life and religious practices [5, 6]. Religious commitment has two dimensions of intra-individual and extra-individual. The intra-individual religious commitment means the value of a person’s religious beliefs and a degree of commitment and loyalty to the religion. The extra-individual religious commitment is said to be relative to the willingness of a person to engage in religious activities [7, 8]. Regarding the role and importance of spirituality in health [9] as well as the role of observance of professional ethics by nurses in improving the health status of patients.

## Main text

### Methods

This study aimed at determining the relationship between religious commitment and moral sensitivity among nurses working in the ICU sections of Ilam and Kermanshah cities in the west of Iran. The present study was a cross-sectional descriptive-analytic study carried out on 110 nurses working in ICU wards of two western cities (Ilam and Kermanshah) in Iran. The entry criteria included having at least a bachelor’s degree in nursing, having a work experience of more than 1 year in the ICU and informed consent for participation in the study.

First, the researchers referred to the ICU wards of the hospitals in the cities of Ilam and Kermanshah after receiving permission from the relevant authorities by referring to three shifts in the morning, evening and night shifts and holidays. The researchers, while explaining the research goals for the nurses participating in the study, obtained their informed consent to participate in this study. Then, the nurses were assured that their participation did not have any harm or loss to them, and the information obtained from these nurses would be reported anonymously. The questionnaire was then provided to the nurses and they were asked to complete the questionnaire when they were willing. Given that the nurses’ fatigue may have prevented them from completing with the questionnaires accurately, they were asked to complete the questionnaire at the time of rest and to have sufficient time. The questionnaires were collected on the next day. The hospitals that were visited in Ilam included ICU’s of Imam Khomeini Hospital, Martyr Mostafa Khomeini Hospital and Ayatollah Taleghani Hospital. Also, in the city of Kermanshah, ICU sections of Ayatollah Taleghani, Imam Khomeini, Imam Reza and Imam Ali hospitals were investigated.

The instrument used includes a demographic questionnaire, religious commitment questionnaire and moral sensitivity questionnaire in nurses. Religious Commitment Questionnaire had 10 items in two dimensions: intra-individual religious commitment (6 questions) and extra-individual religious commitment (4 questions) in the form of 5-point Likert scale, which describes the level of religious commitment of a person [3]. The questionnaire of moral sensitivity in nursing had 25 items, which includes the amount of respect for patient independence, the level of awareness of how to communicate with the patient, the level of professional knowledge, the experience of problems and ethical conflicts, the use of ethical concepts in moral decision-making and honesty and benevolence. A five-point Likert scale was used from zero (no idea) to four (totally agree) and the scores ranged from zero to 50 (low moral sensitivity), 50 to 75 (a moderate moral sensitivity), 76 to 100 (a high moral sensitivity [10, 11]. Data using software spss16 were analyzed using descriptive statistics (mean and standard deviation) and analytical tests (independent t-test, ANOVA and regression).

### Findings

According to the findings, MS and RC rates in female nurses, having MA degree and higher were higher than male nurses with BA degree which was statistically significant. Moreover, with the increase in age and work experience, the nurse MS and RC also increased, which were statistically significant (Tables 1, 2, 3).

**Table 1:** Mean (SD) of religious commitment and moral sensitivity in nurses working in the ICU participating in the research

	Status	N (%)	RC	MS
Sex	Man	47 (42.7)	34.97 (5.64)	53.25 (13.30)
	Female	63 (57.3)	37.42 (3.28)	63.66 (10.15)
	P, F		F = 8.17, P = 0.005	F = 21.67, P = 0.000
Level of education	Below Masters	61 (55.5)	35.57 (5.39)	56.42 (13.26)
	Master of Science and above	49 (44.5)	37.38 (2.75)	62.69 (11.02)
	P, F		F = 4.37, P = 0.03	F = 7.03, P = 0.009
Marital status	Single	64 (58.2)	36.04 (4.71)	57.57 (14.20)
	Married	46 (41.8)	36.84 (4.41)	61.50 (9.82)
	P, F		F = 0.84, P = 0.36	F = 2.60, P = 0.10
History ethics workshop	Yes	62 (56.4)	36.53(3.95)	59.90 (11.44)
	No	48 (43.6)	36.18 (5.33)	58.33 (14.14)
	P, F		F = 0.15, P = 0.69	F = 0.41, P = 0.52

**Table 2:** Mean (SD) religious commitment score and ethical sensitivity with their dimensions in the ICU participating in the research

	SD	Mean
Religious commitment		
Internal religious commitment	23.10	3.10
External religious commitment	13.27	2.65
Total religious commitment score	36.38	4.58
Then respect for patient independence	7.33	3.08
Then awareness of how to communicate with the patient	13.04	3.33
Moral sensitivity		
Professional knowledge later	5.26	1.66
Then experience moral problems	5.76	2.36
Then use ethical concepts in moral decision making	10.57	3.29
Then sincerity and goodwill	17.23	4.65
Total moral sensitivity score	59.21	12.65

**Table 3:** Relationship between RC wite MS

Model	Sum of squares	Df	Mean square	F	Sig.
MS					
Regression	8286.550	1	8286.550	97.572	0.000
Residual	9172.214	108	84.928		
Total	174.58.764	109			

### Discussion

In the study of Baloochi Beydokhti et al. on the nurses’ group of Gonabad city of Iran, it was shown that the amount of internal religious orientation was higher than the external religious orientation and there was a significant weak relationship between the amount of internal religious orientation and the rate of MS [12] which showed the relationship between religion and MS in nurses. In the study of Farahaninia et al. on the nursing students, the implementation of six sessions of the ethical intervention program can lead to an increase in the MS score of nursing students [13], which confirms the role of religious factors in nurses’ MS status.

In this study, the MS mean score for most nurses was average and equal to (M = 59.21) (SD = 12.65). Various studies have been performed on the rate of MS in nurses working in the intensive care units of Iran and their results were compared with the results of this study. In the study of Mohammadi et al. on the nurses working in the specialized care units of South Khorasan province in Iran, the results showed that the MS score of nurses was average and upward with a score of 3.05 (0.68) out of a total of 4. It was also shown that by increasing the MS score, the attitude score toward patient’s rights was also increased [14]. Different studies have been conducted on nurses working in non-specialized care units of Iran.

### Conclusion

According to the results, it is necessary to take necessary religious interventions to improve the rate of RC in nurses so as to provide the necessary field in order to improve the status of MS.

## Limitation

Small sample size.

## Data Availability

Data are available upon request.

## Abbreviation

ICU: intensive care unit
